# Can community health workers play a greater role in increasing access to medical abortion services? A qualitative study

**DOI:** 10.1186/s12905-017-0391-1

**Published:** 2017-05-25

**Authors:** Pallavi Gupta, Sharad D. Iyengar, Bela Ganatra, Heidi Bart Johnston, Kirti Iyengar

**Affiliations:** 1 Action Research & Training for Health (ARTH), Satyam, Ramgiri, Badgaon, Udaipur, Rajasthan 313011 India; 2 UNDP/UNFPA/UNICEF/WHO/World Bank Special Programme of Research, Development and Research Training in Human Reproduction (HRP), World Health Organization, 20 Avenue Appia, 1211 Geneva-27, Switzerland; 3 Swiss Tropical and Public Health Institute, Socinstrasse 57, P.O. Box 4002, Basel, Switzerland; 4 Department of Women’s and Children’s Health, Karolinska Institutet, WHO collaborating Centre, Karolinska University Hospital, SE-17176 Stockholm, Sweden

**Keywords:** India, Medical abortion, Community health workers, Post-a bortion complication, Sterilisation

## Abstract

**Background:**

Despite being legally available in India since 1971, barriers to safe and legal abortion remain, and unsafe and/or illegal abortion continues to be a problem. Community health workers have been involved in improving access to health information and care for maternal and child health in resource poor settings, but their role in facilitating accurate information about and access to safe abortion has been relatively unexplored. A qualitative study was conducted in Rajasthan, India to study acceptability, perspectives and preferences of women and community health workers, regarding the involvement of community health workers in medical abortion referrals.

**Methods:**

In-depth interviews were conducted with 24 women seeking early medical abortion at legal abortion facilities or presenting at these facilities for a follow-up assessment after medical abortion. Ten community health workers who were trained to assess eligibility for early medical abortion and/or to assess whether women needed a follow-up visit after early medical abortion were also interviewed. The transcripts were coded using ATLAS-ti 7 (version 7.1.4) in the local language and reports were generated for all the codes, emerging themes were identified and the findings were analysed.

**Results:**

Community health workers (CHWs) were willing to play a role in assessing eligibility for medical abortion and in identifying women who are in need of follow-up care after early medical abortion, when provided with appropriate training, regular supplies and job aids. Women however had apprehensions about contacting CHWs in relation to abortions. Important barriers that prevented women from seeking information and assistance from community health workers were fear of breach of confidentiality and a perception that they would be pressurised to undergo sterilisation.

**Conclusions:**

Our findings support a potential for greater role of CHWs in making safe abortion information and services accessible to women, while highlighting the need to address women’s concerns about approaching CHWs in case of unwanted pregnancy. Further intervention research would be needed to shed light on the effectiveness of role of CHWs in facilitating access to safe abortion and to outline specific components in a programme setting.

**Trial registration:**

Not applicable.

## Background

In India, abortion has been legal since 1971 for a wide range of indications such as risk to the woman’s life, grave injury to her physical or mental health, failure of contraception, or substantial risk that the child may be born with serious physical or mental abnormalities, yet many barriers prevent women from accessing safe and legal abortion [1, 2]. Most safe abortion facilities are concentrated in cities, while rural areas severely lack abortion facilities [3]. It is estimated that approximately two-thirds of all abortions in India are performed by unauthorized or unskilled providers [4], and that unsafe abortion is responsible for an estimated 8% of maternal deaths in India [5].

Medical abortion using mifepristone and misoprostol pills is a safe, effective technology suitable for provision at the primary care level [6], and hence has the potential to expand access to safe abortion to rural areas. It is still not widely available, and even where available, barriers such as lack of information to women on its availability, or service delivery guidelines that require women to make multiple visits to a clinic, might pose significant barriers for women living in rural areas in accessing medical abortion [7].

Community health workers facilitate or provide a wide variety of health related services successfully in different countries including maternal care, immunization, management of childhood illnesses, infectious or chronic non-communicable diseases, information about and/or distribution of contraceptives. [8–13]. In facilitating access to safe abortion care, community level workers can provide information about safe providers, and provide referrals or accompany women to safe and legal facilities [14, 15]. An operations research study conducted in Nepal found that female community health volunteers were effective in informing women about medical abortion, conducting urine pregnancy tests, referring women to a safe place for abortion, and providing post-abortion contraceptive counselling [16].

Under the National Rural Health Mission (NRHM), in 2005, Government of India introduced a cadre of female community health workers, called the Accredited Social Health Activists (ASHAs) at village level. ASHAs’ main responsibilities feature facilitating community access to health care and health facilities, and include accompanying women for antenatal care and delivery; accompanying children for immunization, providing information on family planning methods and distributing them, care for minor ailments and conducting urine pregnancy tests; providing postpartum maternal and newborn care. While ASHAs have been provided with urine pregnancy test kits, information on whether women with unwanted pregnancy are linked to abortion services on the basis of the test result is not available [17, 18]. As per the law in India that governs provision of induced abortion, CHWs including ASHAs are not authorized to provide abortion services, including administration of medical abortion drugs. Further, the basic education and job specific training of CHWs is very limited, and it does not favour exploring provision of medical abortion as a potential role for CHWs.

A recent study in three countries assessed the performance of CHWs in facilitating women’s access to safe abortion. Specifically, the study assessed if CHWs could accurately identify women who are eligible for medical abortion and can identify women who are in need of follow-up care after medical abortion [19]. As part of the clinical study, women who came to avail abortion at identified health centres were assessed by a CHW and a clinician. The eligibility toolkit included a calendar, a pregnancy test kit, a gestational age wheel and a checklist of contraindications to medical abortion in Hindi language. The follow-up toolkit included a checklist of indications that could represent a complication. The study in India was implemented in Rajasthan, in partnership with a non-government organization, Action Research & Training for Health (ARTH), Udaipur. Overall the study showed a moderate to high level of agreement between CHWs and clinicians in assessment of eligibility depending on CHWs’ basic level of education and prior experience. In India, where the basic level of education of CHWs is low (8–10 years of education), CHWs were moderately effective (80% accuracy) in assessing women’s eligibility for medical abortion.

Hence, to improve women’s access to safe abortion, increasing CHW involvement appears to be a promising strategy. However, at present we do not know if the strategy of CHW providing abortion information would be acceptable to the primary stakeholders - the CHWs and women in their communities. Hence, for CHWs to be involved in a programme setting, it would be important to understand perceptions of CHWs and women regarding an expanded role for CHWs.

The aim of this qualitative study was to explore the acceptability, perspectives and preferences of women and community health workers regarding involvement of community health workers in medical abortion referrals and follow-up.

## Methods

Study setting: This qualitative study, conducted in 2013, was implemented in Udaipur district of southern Rajasthan, India. Eighty percent of population of district of Udaipur lives in rural areas, and only 39% women are literate [20], with only 19% households having a motorised personal transport in 2007–08 [21].

Women participating in the clinical study described above, that assessed if CHWs could accurately identify women’s eligibility and need for follow-up care for medical abortion, were purposively sampled for the qualitative study. Recruitment of women for qualitative study started after four months of  implementation of the clinical study. Women were selected from three health centres, two of which are located in rural areas and one in the city. The rural health centres provide only first trimester abortion service twice a week when a gynaecologist visits the health centres, while the urban centre provides abortion services daily. All three study health centres were managed by non-governmental organisations.

In-depth interviews were conducted with 24 women seeking abortion services at either one of the three study centres. In addition, 10 community health workers who were part of the clinical study described above were also interviewed [19]. Of the 24 women interviewed, 12 had participated in eligibility assessment and 13 had participated in follow-up assessment (one of the women had participated in both eligibility and follow-up assessments). Respondents were selected so as to ensure diversity with respect to residence (rural/urban), number of children, age, caste and education.

Six of the ten participating CHWs were government appointed ASHAs (henceforth referred to as ASHAs) while four were village health workers (VHWs), appointed by the non-government organization (henceforth referred to as VHWs); when referring to both ASHAs and VHWs as one entity, we use the term community health workers, or CHWs). ASHAs had eight or more years of education and 20–30 days of specific health training provided by the government. The VHWs had five to eight years of school education and 28 days of health training provided by the NGO. For the purpose of the clinical study, both cadres underwent training of six days on abortion related issues and use of assessment checklists. In this qualitative study, from the national policy perspective, we explored women’s interaction only with ASHAs, because ASHAs are appointed by government health agencies, throughout the country. We included VHWs in the study for the limited purpose of highlighting the performance of community level workers who have an optimal supportive mechanism in terms of skill supervision, supplies and no pressure to meet family planning targets, unlike ASHAs.

Research assistants sought consent from women to participate in in-depth interviews on the day of receiving Mifepristone, after they had been assessed by both CHWs and clinicians. Consenting women were interviewed at the health centres (except for two women who preferred to be interviewed at home), at a time convenient to them, within a fortnight of the initial visit to the health centre. After details of the study were fully explained, written consent was obtained from all the study participants for interviews and separately for audio recording. Illiterate respondents put their thumb impression in the presence of a witness. All but two respondents agreed to audio recording.

Semi-structured interview guides were developed for women as well as for CHWs after pilot testing. Each respondent was interviewed once; the interviews lasted around 45–60 min.

Four female interviewers conducted interviews between August and November 2013, in Hindi or in the local dialect, (Mewari), in a private space and maintained confidentiality.

All interviewers were postgraduates in social sciences, with years of experience of working on issues of reproductive health, and had received additional training. The research team prepared verbatim transcripts with the help of audio recordings and after every two to three interviews, reviewed them to identify issues to be further explored, and discussed ways to elicit more in-depth information. We coded the transcripts using ATLAS-ti 7 (version 7.1.4) in the local language and generated reports for all codes, identified emerging themes and analysed findings.

We first present findings from CHW interviews, organized into three broad thematic areas- perceptions about their current roles especially in relation to unwanted pregnancies; experiences of the project and learning to play a greater role in facilitating medical abortion services; and perceptions about playing a greater role in assessing eligibility, follow-up care and arranging referrals. This is followed by women’s perceptions on current roles of CHWs, their past experiences with CHWs, and their views on greater involvement of CHWs in facilitating abortion care.

The Institutional Ethics Committee of the local non-government organisation, Action Research & Training for Health (ARTH), Udaipur approved the study.

## Results

### Profile of community health workers

Most of the ten female CHWs who participated in the study were married and had children. Six were educated up to or above class ten. Most had been working as CHWs for at least five years, with two being two years into the role.

### Views of community health workers

#### CHW’s views on their current roles

Government appointed CHWs (ASHAs) reported their main roles as facilitating maternal care, conducting meetings of adolescent girls, weighing children, accompanying children for immunization, and maintaining records at the *anganwadi* (pre-school child care centre in the village run by the government). Three ASHAs mentioned distributing oral contraceptive pills and condoms, accompanying women for sterilisation and helping in case of other gynaecological problems.

All NGO appointed CHWs (Village Health Workers, VHWs) reported distributing condoms, oral contraceptive pills, emergency contraceptive pills, iron folic acid tablets, conducting pregnancy tests, postpartum home visits, and adolescent girls’ meetings. They also reported accompanying women for abortion, injectable contraceptives, antenatal care or delivery at the health centre. The VHWs reported meeting twenty to twenty-five women each with unwanted pregnancy in the twelve months prior to the study.

Only two ASHAs spontaneously mentioned conducting pregnancy tests and helping women with unwanted pregnancy but when specifically asked about their role in case of unwanted pregnancy, all replied that women in such a situation do contact them. Each reported having met three to ten women with unwanted pregnancy in the twelve months prior to the study. They said that if required they conduct a pregnancy test and help such women seek abortion services.

- *“Those (women) who miss their periods and do not want to continue (the pregnancy), they come with lot of tension… what to do, where to go? Then I take them to (ARTH) health centre if they are up to two months (of pregnancy). If (the pregnancy is) around three months, then I take them to Surajpol (urban centre).*” (P3, an ASHA working in the urban area)

Most CHWs felt that women trust them and approach them without any hesitation as they have been working in the area for a long time. CHWs were of the opinion that uneducated women, or those who have not had a prior abortion experience, or those with little exposure were more likely to take their advice.

- *“… (If) she is educated, has the information then what does she have to do with us (she doesn’t consult us). Those who live in Surat, Mumbai, they have all the information, they don’t even ask us…someone knows and has money, then they go direct to Udaipur…one who is very poor, not educated, she waits for us, she will come (to us)…”* (P10, a VHW working in the rural area)

Most of the ASHAs said that they accompany women to the hospital for abortion. On the other hand, VHWs said that although they do accompany women for abortion, some women do not want to be seen being accompanied by them in the village since it raises suspicion among people, and they ask the VHWs to meet them directly at the health centre.

- *“I do the test and tell her whether she is pregnant or not. If (she is) pregnant, then (I tell her), let us go to the hospital, let us talk to the doctor, how many months is it. I go along to the hospital, they (women) do not know the place, I go with every patient”* (P1, an ASHA working in the urban area)

- *“…they feel if there is someone with them, then people will see and wonder where are the two going together, therefore they do not want to be seen going out of the village with someone. Instead they ask me to meet them somewhere on the way or at the health centre…”* (P5, a VHW working in the rural area)

All ASHAs said that they do not take money from women for abortion referral, though women sometimes pay for their travel or compensate for the entire day spent with them. However, when asked why some women with unwanted pregnancy might not be contacting the ASHAs, two of them replied that perhaps women think that they would have to spend more money if they take the ASHA’s help.

Four out of the six ASHAs interviewed, held the opinion that abortion is not ‘good’ and even referred to it as ‘sin’, especially if it was the woman’s first pregnancy or beyond a certain gestation period, like two months. They thought it was justified if the couple had two or more children and did not want any more.

- *“A woman had got abortion done twice without asking me. This time she came to me for a third abortion, I refused, told her that you have already committed this sin twice, now you want to do it another time…”* (P7, ASHA working in urban area)
- “*I accompany if the time is less, if it is up to two months, then I go along. If it is three or four months, then I tell them where to go, I don’t go along…”* (P2, ASHA working in urban area)

Have community health workers come in contact with women after an abortion?

In order to ascertain the relevance of community-based follow-up care after medical abortion, we tried to understand whether CHWs come in contact with women after their abortions. Almost all CHWs, working in both rural and urban areas, reported meeting at least one woman with post-abortion complications in the past one year, with rural CHWs having met more such women. The most common situation that all the CHWs had encountered was of women taking tablets over the counter from a pharmacist, and then seeking their help for post-abortion complications. CHWs mentioned that women prefer taking tablets from pharmacists or local unqualified providers because of fear of being recognised at a health facility, health facilities asking for a guardian if the woman looked young, and travel time to reach a formal health facility.

VHWs reported that sometimes they encounter problems while helping women access abortion services.

- *“I did her (pregnancy) test and counselled her to go to the health centre…however, instead she took tablets from somewhere and developed complications…her mother-in-law abused me so much and held me responsible for giving her wrong medicines…” *(P6, VHW working in rural area*)*
- *“I tested a woman in the village, test was positive…she took one tablet from the health centre but did not come back for the second dose…when I met her again in the village she shouted at me that she had been given wrong tablet at the health centre and had lot of problem because of that…she said she had nearly died…” *(P10, VHW working in rural area)

CHWs’ experience of the project and learning to play a greater role in facilitating medical abortion services.

All CHWs interviewed in this study were exposed to six-day training on abortion related issues (as part of the clinical study on assessment of eligibility), for example, laws related to abortion, methods of abortion, need to maintain confidentiality, confirmation of pregnancy, use of gestational age wheel, and use of eligibility and follow-up checklists. ASHAs particularly appreciated the opportunity to learn more about medical abortion as their government training had not covered it in such detail, e.g. how medical abortion tablets work, legal issues around abortion, calculating the date of last menstrual period (LMP), possible complications due to unsafe abortion, how to know whether abortion is complete and whether woman needs follow-up. A couple of ASHAs mentioned learning about contraindications and another few said that they learnt the importance of maintaining confidentiality.

- *“Earlier, we all used to take the women and get the surgical (abortion) done. We used to take women to the doctor who put instruments inside and took it out and cleaning was done. That is what we knew.”* (P1, ASHA working in the urban area)

- *“…earlier we used to discuss with each other about women who sought abortion, but in the training we learnt that we should not talk like that and keep the matter between the woman and ourselves.., speak in the way that we can win their confidence so that if they have any problem they first come and share that with us and only then take action…”* (P3, ASHA working in the urban area)

When asked about specific components of the toolkit, they mentioned that the calendar and gestational age wheel were new for all the CHWs and they found them very useful. They said that now they could quickly and easily assess whether a woman is eligible for MA and accordingly counsel her.

- *“… (Earlier, I) did not know how to calculate the exact date...now if the woman tells us in terms of the local calendar (based on the position and sighting of the moon), then we can find out the LMP using the calendar we got during training…”* (P1, ASHA working in the urban area)
- *“…With this (Gestational age wheel) within a second, we put the date and immediately tell how many weeks pregnant she is, while the woman is still in front of us, and without using a pen or anything”* (P3, ASHA working in the urban area)

ASHAs also appreciated the hands-on practice that they got as against their usual training which is often theoretical.

- *“…immediately after the training we were made to do a practical. Most of our other trainings happen, there is no practical…then when we work in the village we remember some and forget some. But here with simultaneous practice we realized what we needed to learn more and there itself could learn and improve…”* (P8, ASHA working in the rural area)

What do CHWs feel about conducting eligibility assessment in the community?

All the CHWs felt that earlier too, they had helped women access abortion, but now they would be able to do that job better. Using the checklist at the community level, they could assess whether the woman is eligible for medical abortion, or might need surgical abortion. They felt that this would help them to counsel women better, and reassure her that she need not be scared and that she could get an abortion using pills.

According to one of the CHWs, since many facilities only provide first trimester abortions, women would be able to go to the health facility knowing whether they will get the abortion service at that facility or not.

- *“Many such women come to the health centre, who are told after examination that their gestation is more than three months and they cannot be provided abortion here (at that centre), then they have to go back…”* (P6, VHW working in the rural area)

One apprehension that many CHWs shared was about women not knowing the date of their last menstrual period correctly, on which their entire assessment depends. Thus if the women tell them the wrong LMP, the gestational age that they calculate would also be wrong and the CHW could draw a wrong conclusion.

CHWs working in the rural area shared that while they feel confident to do this work, it might be difficult to find a private place to talk. They were also a bit concerned that a woman found eligible for medical abortion by the CHW might be found ineligible by the doctor, which might later become a source of tension between them.

- *“I can do this but if someone from their family is around, if a woman’s husband or mother-in-law are there, she may feel shy of them and she may not talk in front of them. Then it would be difficult, otherwise no problem, I can do it”* (a VHW working in rural area)

Potential for providing follow-up care in the community

All CHWs felt that women do not like to go to the health-facility for follow-up after medical abortion because of financial and time constraints and fear of arousing suspicion in the family due to repeated visits. All CHWs felt that women would prefer to talk to them and would be comfortable in doing so as over the years women have developed trust in them.

However, almost all of them said that they would not like to take the sole responsibility upon themselves and would prefer to ask women to consult the doctor who could conduct a physical examination. They felt that there could be some internal complication which they might not be able to identify on the basis of a checklist. Two VHWs also reasoned that the doctor could better motivate women to use a long acting contraceptive like copper-T or injectable, thus preventing the need for repeat abortion.

### Profile of women seeking abortion

Eleven of the twenty four women were rural, all were married and majority had 1–2 children. More than half the women had five years or less of formal education (Table 1).

**Table 1:** Profile of women seeking abortion

Age (*n* = 24)		
	<20	1
	20–24	6
	25–29	11
	30 & above	6
	Mean age	26 years
	Range	18–40 years
Caste^a^ (*n* = 24)		
Scheduled caste		1
Scheduled tribe		12
Other castes		11
Number of living children		
	None	3
	1 to 2	14
	3 to 4	6
	5 or more	1
Education		
	Illiterate	9
	1st to 5th	5
	6th to 8th	1
	9th and above	9
Marital status (*n* = 24)		
	Married	24
Abortion service availed at (*n* = 24)		
	Rural centre 1	7
	Rural centre 2	1
	Urban centre 1	16^b^

^a^Scheduled castes and tribes represent socio-economically marginalized communities in India

^b^Three women who were interviewed at the urban centre lived in villages more than 25 km from the urban centre, hence they were categorised as ‘rural’. Thus, out of the total twenty-four women interviewed, eleven were categorised as living in rural areas. Of the eleven rural women, six had participated in eligibility assessment and five in follow-up assessment. Amongst the thirteen urban women, eight had participated in eligibility assessment and five in follow-up assessment

### Views of women seeking abortion

#### Do women know about community health workers?

All women knew about the ASHA but only eleven of them had ever met an ASHA. Those who had interacted with the ASHAs in the past had done so either in relation to antenatal check-up, delivery, children’s immunisation or for obtaining oral contraceptive pills.

#### Women’s views on current role of community health workers (ASHAs)

Women reported the main roles of ASHAs were related to delivery and calling children for immunisation. A few of them also mentioned that ASHAs conduct meetings of adolescent girls and women, weigh children and administer polio drops.

- “*Someone comes to ask for small children but since we do not have small children so now (she) does not come to our house.”* (P18, urban non-tribal woman)

Nearly a quarter of them said that the ASHAs give oral pills and condoms and almost a third said that they motivate and accompany women for sterilisation. Although very few women mentioned spontaneously that ASHAs conduct pregnancy tests or help women to seek abortion, 6 out of 23 women said, upon probing, that ASHAs conduct a urine pregnancy test when contacted by a woman with unwanted pregnancy (one of the women participants was herself an ASHA, hence this question is not applicable to her). However, only three of them said that she helps women with unwanted pregnancy to get abortion.

#### Whether women sought help from CHWs in seeking abortion services?

Of 23 women participants who came to the health centres for termination, only three had contacted the ASHA about their unwanted pregnancy prior to reaching the centre (excluding the one who was an ASHA herself). These 3 women were urban, of whom two took the pregnancy test from ASHAs, and one was referred to the health centre. Most women had heard about the centre from a relative, friend or neighbour, or had accompanied someone to that centre in the past or were referred by chemists or local private practitioners from whom they had bought the pregnancy test kits. None of the respondents who reached the health centre were accompanied by an ASHA.

The reasons women gave for not contacting the ASHA in case of unwanted pregnancy included a perception that they would be pressured to undergo sterilisation, fear of being scolded, concerns about confidentiality and the possibility of being directed towards providers who charge more money.

- “I*f I talk to her about abortion, then she asks me to get both abortion and sterilisation done. But my husband does not agree for sterilisation…she was once taking me for sterilisation forcefully, just then my husband arrived and said that he will not let me go for sterilisation. So, we do not talk to anyone at the anganwadi about such things…* (P16, rural tribal woman)

- *… (I) do not tell her (ASHA) about this. If I tell her then she may tell someone else…things become public…so (I) do not tell anyone…”* (P9, an urban non-tribal woman)

- *“…I know that she does urine test, women go to her…but what if she tells someone in my family, I have two older sisters-in-law, if they come to know and tell my father or brother. That is why I am scared, so I do not talk to the ASHA about it....”* (P21, rural non-tribal woman)

Some women said that they did not know that ASHAs could help women with unwanted pregnancy

- *“I did not know that such a thing (pregnancy test) is done at the anganwadi, now that you are telling me, I am learning about it…”* (P20, an urban non-tribal woman)

#### Women’s views on involving CHWs in assessment of eligibility for medical abortion

When respondents were asked about their views on ASHAs assessing women’s eligibility for medical abortion in the community, views of rural and urban respondents seemed to differ. Four of the six urban abortion clients, were in favour of ASHAs playing a greater role, and said that if ASHAs were to do such work in the community, women would go to the health centre with more confidence. One respondent felt that the ASHA would talk to her in greater detail, while she felt hesitant talking much to the doctor.

- *“It will be good, this is ASHAs’ daily work. Women will benefit, will go with more confidence…do not see any problem, ASHA does not take any money for it…”* (P3, an urban non-tribal woman)
- *“It will be beneficial, the doctor talks only main things. (I) can talk openly with the ASHA… (I) feel scared of the doctor…just answered whatever the doctor asked as yes and no, (On the other hand, I) can talk more freely with the ASHA…see no disadvantage of it, women will trust the ASHA*” (P5, an urban non-tribal woman)

The remaining two urban respondents had not interacted with ASHAs in the past and preferred to consult the doctor rather than rely on ASHAs. Interestingly, one of them said that those who do not know where to go may contact the ASHA for information or to have them accompany.

- *“…if the woman has not gone there (to the hospital) earlier, then she will take the ASHA along and if she has gone there before, then why will she ask the ASHA? She will go to the doctor on her own…”* (P1, an urban tribal woman)

Amongst the six rural abortion clients who were interviewed regarding the role of ASHAs in eligibility assessment in the community, five had reservations about it. They reasoned that ASHA is not as skilled as a doctor, she could not actually conduct an examination nor would give her medicines, and they also feared breach of confidentiality.

- *“It will be beneficial only if (I) come to the hospital. I don’t know what good (it) will be meeting her, she will only talk…”* (P8, a rural tribal woman)
- *“How can we tell the ASHA? She lives in the village itself, what if she tells someone else…doctor is at the health centre and will then go to her house…”* (P9, a rural non-tribal woman)

Only one rural respondent was in favour of ASHA conducting an eligibility assessment in the village so that she would not have to go repeatedly to the health centre.

- *“(If ASHA does the eligibilty asessment in the village itself) The advantage is that we will not have to come so far, not spend money on travel. Yesterday both of us (me and my husband) spent the whole day on this, today too we have come and have to again come day after tomorrow…”* (P6, a rural tribal woman)

#### Should community health workers assess the need for follow-up at home?

Many respondents were of the view that it will be good if they do not have to go for a follow-up visit or if ASHAs can inform them whether they need to go back for a follow-up visit. Women cited many problems in coming to the health centre for follow-up visit, for example, lack of time to complete household work before leaving for the health centre, money spent on travel or having to give explanations to other family members, as illustrated by the following examples:

- *“…it will be good if ASHA can tell us at home (whether follow-up visit to the centre is needed), money on travel will be saved and I can take care of household chores. Today my husband had to go to work without food, since I didn’t have time to cook in the morning…”* (P 17, a rural tribal woman)

One respondent mentioned that her husband lost the day’s wages since he had to accompany her. Two other respondents mentioned that they had to make excuses to come to the health centre.

- *“… (If ASHA is able to assess the need for follow-up at home, then), I would not have had to come here, will not have to take so much trouble. (Today) I used the excuse of getting my older child treated…I had to lie to my mother-in-law and father-in-law, spent so much money in travelling and also wasted time…”* (P22, an urban non-tribal woman)

At the same time, many respondents said that they would not trust the ASHA’s assessment since she is not sufficiently trained and because she could not conduct physical examination. One woman, who has been under pressure for sterilisation from the ASHA, in fact very emphatically stated that there would not be any benefit of ASHA conducting the assessment.

- *“… (it) would not have been of any use…I will come for examination,…she will only talk , not do any examination, she will only say from her mouth (verbally) that your work is done but those who examine are here (in the centre)…I have to come here, here examination is done by making one lie on the bed…”* (P16, a rural tribal woman)

## Discussion

Our study turns up mixed results regarding the acceptability of expansion of community health workers’ role in the medical abortion process. CHWs (both ASHAs and VHWs) viewed this as part of the work they were already doing and were enthusiastic about the tools and training they received. They felt they could take on greater roles in facilitating access to medical abortion care and were confident about it. Women on the other hand, reported lesser contact with CHWs when faced with unwanted pregnancy, because of concerns such as pressure to also undergo sterilisation, fear of breach of confidentiality, being scolded, or directed towards providers who charge more money. Also, some women did not know that ASHAs could help them in such a situation. However, many women also saw benefit in being guided by CHWs, such as saving time and money by not having to go repeatedly to the health centre.

In our study, ASHAs were enthusiastic about taking on greater roles in enabling women access safe abortion services. Other studies in India also identified ASHAs’ desire to acquire more health-related skills [8]. Studies have shown that women and men in India lack information on the legality of abortion and have shown preference for medical abortion [22–24]. When women are unsure about whether a health centre would give them a choice between surgical and medical methods, and whether they are eligible to receive medical abortion, they might approach informal providers [22, 25, 26]. In India, there is a huge gap between the number of reported abortions and actual mifepristone sales, and that could be a reflection of various barriers that women face in accessing safe legal abortion services, as well as lack of reporting even when safe abortions are provided [24].

CHWs are well positioned to play a role in reducing some of those barriers by guiding women with unwanted pregnancies to safe abortion providers, assessing their eligibility for medical abortion and reducing the need for follow-up visits after medical abortions, and thus improving women’s access to safe abortions.

There was contradiction between abortion clients’ views and ASHAs’ views on whether women with unwanted pregnancy contact ASHAs before seeking abortion services. While many of the women were not aware that ASHAs could help them with unwanted pregnancies and none of the women were accompanied by the ASHA, ASHAs seemed to suggest that most women contacted them. This contradiction could have emerged from two reasons- first, ASHAs may have overstated their role, perhaps only a minority of women contacted ASHAs when faced with unwanted pregnancy. Second, almost half of the abortion clients in our study were urban and thus had greater awareness of abortion facilities.

A few women in our study had reservations about approaching ASHAs, mainly because they feared pressure for sterilisation or breach of confidentiality. Despite Government of India’s commitment to a “target free approach”, results of this study highlight the challenges women face not only in accessing abortion but also in accessing family planning methods of their choice, with ASHAs pressurising them to undergo sterilisation. Other studies have also pointed to pressure on frontline health workers to achieve sterilisation targets [27–29]. Another significant reason for lack of trust in ASHAs relates to the perception that they might not maintain confidentiality, especially expressed by rural women, a greater proportion of who had interacted with ASHAs. A study conducted in the southern Indian state of Tamil Nadu documented the high priority women placed on confidentiality and privacy while seeking abortion services [30]. Our findings suggest that CHWs’ own views and values about abortion might also affect the manner in which they respond to women in need of abortion. Hence, for the community to trust them and for ASHAs to provide reproductive health related services effectively, it would be necessary that community health workers are trained to provide non-judgemental services in a confidential manner. However, the most crucial step would be that frontline workers and CHWs are freed from pressure to achieve sterilization targets, so that they can provide balanced information about all available contraceptive methods.

In our study, most women who had sought services from ASHAs had done so in relation to antenatal care, delivery, children’s immunisation, contraceptives, etc. As in our study, it has also been documented by other studies, that ASHAs spend most of their time on providing maternal and child health services including antenatal care and delivery- the activities that contribute to most of their incentives, and that their focus on family planning services is weak [31]. A focus on select services often keeps women with other needs such as abortion out of the ASHA’s purview. It would therefore be essential for the health programmes to prioritise pregnancy testing and referrals for abortion as an important task of ASHAs. The fact that the NGO appointed VHWs had helped more women with unwanted pregnancy implies that if provided a supportive work environment and freedom from meeting service specific targets, CHWs can provide better and wider range of services to women.

In our study, some women expressed that CHWs could guide them on the need for a follow-up visit. Hence it appears that CHWs providing follow-up care after medical abortion is possible, and might address financial, time and confidentiality constraints related to repeatedly visiting a facility. For example, a series of questions posed to women in South Africa using mobile technology showed that this was very helpful in reducing women’s anxiety [32]. In the Indian context where women’s literacy levels and access to mobile phones may be a constraint, CHWs could well perform such a role in following up women after abortion. However, one important precondition for women to inform CHWs about their abortions, would be that CHWs maintain confidentiality, and that women trust them.

The ASHAs found the practical training received as part of the study very useful and also appreciated the toolkit as it reduced their dependence on auxiliary nurse midwives to calculate the date of last menstrual period. A few of them specifically appreciated learning about the importance of maintaining confidentiality, this corresponds with the reasons that women gave for not seeking services from them. Lack of proper training of ASHAs was pointed out in another study from India that identified gaps in both quantity and quality of their training [30]. Good quality practical hands-on training imparted to CHWs and focus on confidentiality will increase their self-confidence and improve the services they provide in the community. Providing simple easy to use job-aids as was done in this study, is an equally critical aspect unlike the almost 300 pages of dense text given as reading material to ASHAs during their stipulated 23 day training period. Furthermore, ASHAs also mentioned constraints in supply of urine pregnancy test kits. Problems of stock-outs of pregnancy test kits and other essential supplies have also been reported [33]. The CHWs’ lack of confidence in their own skills and the supply constraints might further add to the community’s lack of confidence in them [34–37].

Women’s own information on contraception and safe abortion also needs to be increased. Given that mass media approaches to improve information about abortion services are likely to face opposition, interpersonal approaches are more likely to be successful. In an intervention in the same geographical area, pregnancy testing was used as an entry point for CHWs to contact women, and based on their reproductive intentions, to guide them to appropriate services [38].

One limitation of our study was that we only contacted those women who successfully reached a safe abortion centre. Studies and our experience of working in this area have shown that not all women with unwanted pregnancies might be able to reach a safe abortion centre due to multiple socio-economic or health system barriers. It is likely that women included in our study were somewhat more aware than those who could not reach a safe abortion centre. Pathways of contact for women who could not access safe abortion services would have shed greater light on the potential role of CHWs in facilitating safe abortion. Further, we could not explore women’s views regarding VHWs, who represent non-governmental CHWs, were free of sterilisation targets and were better trained on issues such as confidentiality. This was because VHWs were present only in a part of the study area where the NGO operated a field program. While recruiting women for interviews, we did not screen them for the presence of VHW in their village since the focus of the study was to understand women’s interaction with government appointed ASHAs, who were present in all the villages and represent a country-wide cadre. Therefore to maintain consistency, we did not ask women about their experience with VHWs.

## Conclusion

Findings of our study suggest that there is potential for a greater role on part of CHWs in enabling women’s access to safe abortion services. With regards to unwanted pregnancy, CHWs could contact women at multiple points - contraception, pregnancy testing [38], guiding women with unwanted pregnancy, conducting her eligibility assessment for medical abortion, or contacting women after abortion for assessing the need for a follow-up visit. However, whether women contact CHWs before or after their abortions depends to a great extent on the level of trust that women have in CHWs.

Improving the overall working conditions of CHWs through effective training, provision of useful job-aids, and ensuring provision of regular supplies of consumables would be important. Their training needs to add focus not only on contraception and abortion related issues, but must also emphasize confidentiality, reproductive rights and values clarification. Furthermore, for such a programme to be successful, it would be crucial that CHWs are free from pressure to achieve sterilisation targets, formal or informal. It would enable them to provide balanced counselling to women, and to gain their confidence. Better linkages between health centre providers and CHWs could also help. Further, we recommend strengthened monitoring of CHW programmes that goes beyond maternal and child health outcomes, and includes numbers of women offered pregnancy tests, or referred by CHWs for safe abortions, and the quality of contraceptive counselling provided by them.

Health programmes should consider intervention research which is based on a pilot intervention that allows CHWs to play a greater role in facilitating utilization of reproductive health services, including safe abortion. Such a pilot intervention should include elements of training, skill building, lack of pressure to achieve targets and stronger linkages with the health system. Such research should also document the care seeking pathways of women who contacted CHWs but did not reach a safe abortion centre. Based on the results of our clinical study (40), and studies on follow-up after medical abortion [32, 39], the tools and approaches for eligibility assessment and follow-up care could also be further refined. Such research would shed light on the effectiveness of CHWs in facilitating access to safe abortion and outline different components of such an intervention.

## Abbreviations

ASHA: Accredited Social Health Activist
CHW: Community Health Workers
LMP: Last Menstrual Period
NGO: Non-Government Organisation
NHM: National Health Mission
NRHM: National Rural Health Mission
VHW: Village Health Worker
WHO: World Health Organisation
